# A Machine Learning Approach for Detecting Idiopathic REM Sleep Behavior Disorder

**DOI:** 10.3390/diagnostics12112689

**Published:** 2022-11-04

**Authors:** Maria Salsone, Andrea Quattrone, Basilio Vescio, Luigi Ferini-Strambi, Aldo Quattrone

**Affiliations:** 1 Institute of Molecular Bioimaging and Physiology, National Research Council, 20054 Segrate, Italy; 2 Sleep Disorders Center, Division of Neuroscience, San Raffaele Scientific Institute, 20127 Milan, Italy; 3 Institute of Neurology, Magna Graecia University, 88100 Catanzaro, Italy; 4 Neuroimaging Research Unit, Institute of Molecular Bioimaging and Physiology (IBFM), National Research Council (CNR), 88100 Catanzaro, Italy; 5 Biotecnomed S.C.aR.L., c/o Magna Graecia University, G Building, lev.1, 88100 Catanzaro, Italy; 6 Sleep Disorders Center, Vita Salute San Raffaele University, 20132 Milan, Italy; 7 Neuroscience Research Center, Magna Graecia University, 88100 Catanzaro, Italy

**Keywords:** heart rate variability, idiopathic REM sleep behavior disorder, machine learning, classification

## Abstract

**Background and purpose:** Growing evidence suggests that Machine Learning (ML) models can assist the diagnosis of neurological disorders. However, little is known about the potential application of ML in diagnosing idiopathic REM sleep behavior disorder (iRBD), a parasomnia characterized by a high risk of phenoconversion to synucleinopathies. This study aimed to develop a model using ML algorithms to identify iRBD patients and test its accuracy. **Methods:** Data were acquired from 32 participants (20 iRBD patients and 12 controls). All subjects underwent a video-polysomnography. In all subjects, we measured the components of heart rate variability (HRV) during 24 h recordings and calculated night-to-day ratios (cardiac autonomic indices). Discriminating performances of single HRV features were assessed. ML models based on Logistic Regression (LR), Random Forest (RF) and eXtreme Gradient Boosting (XGBoost) were trained on HRV data. The utility of HRV features and ML models for detecting iRBD was evaluated by area under the ROC curve (AUC), sensitivity, specificity and accuracy corresponding to optimal models. **Results:** Cardiac autonomic indices had low performances (accuracy 63–69%) in distinguishing iRBD from control subjects. By contrast, the RF model performed the best, with excellent accuracy (94%), sensitivity (95%) and specificity (92%), while XGBoost showed accuracy (91%), specificity (83%) and sensitivity (95%). The mean triangular index during wake (TIw) was the best discriminating feature between iRBD and HC, with 81% accuracy, reaching 84% accuracy when combined with VLF power during sleep using an LR model. **Conclusions:** Our findings demonstrated that ML algorithms can accurately identify iRBD patients. Our model could be used in clinical practice to facilitate the early detection of this form of RBD.

## 1. Introduction

Rapid eye movement (REM) sleep behavior disorder (RBD) is a parasomnia characterized by the loss of muscle atonia and abnormal behaviors during REM sleep, often as dream enactments, causing injuries [1]. The diagnosis is based on the clinical history in combination with video-polysomnography (PSG) confirmation of REM atonia loss during sleep. The prevalence remains unknown, which is probably caused by the difficulties related to performing gold-standard PSG diagnosis. RBD may be categorized as idiopathic (or isolated iRBD) in the absence of other neurological signs and symptoms or as symptomatic (secondary) when associated with antidepressant drugs or with neurological diseases. In the last decades, increasing scientific interest has been focused on the idiopathic form of RBD. Indeed, although iRBD is formerly a parasomnia, it is now recognized as the prodromal stage of an α-synucleinopathy [2]. This is due to the high risk that patients with iRBD have of developing α-synucleinopathies, such as Parkinson disease (PD), PD dementia, dementia with Lewy bodies or multiple system atrophy [2,3]. Emphasis is now placed on the early detection of iRBD, as the prodromal phase of these disorders, for the opportunity that this creates for timely neuroprotective treatments.

At present, the screening of iRBD patients is performed using the RBD Single-Question Screen (RBD1Q), a screening question for dream enactment with a simple yes/no response, validated in relation to gold-standard PSG diagnosis [4]. RBD1Q was able to detect RBD in a clinic-based cohort of RBD patents and controls with a high sensitivity (94%) and moderate specificity (87%) [4]. As reported by the same authors, the moderate specificity of the RBD screening test was related to several criticisms. Firstly, RBD1Q does not screen for subtle clinical manifestations including sleep talking or sleep yelling, thus excluding these patients from the RBD screening; secondly, patients with non-REM parasomnias can respond positively to the screen [4]; finally, there is lack of validation in a large cohort of iRBD patients and controls.

In the context of emerging tools for RBD screening, heart rate variability (HRV) analysis, a simple and non-invasive measure of cardiac impulses, has been demonstrated to be useful in accurately differentiating patients with PD associated with RBD from those without RBD [5]. Little evidence, however, is present in the literature exploring HRV parameters in iRBD. In addition, reports analyzed only short time intervals (5 min R-R) in small samples of patients [6,7,8]. An Artificial Intelligence (AI) approach based on deep neural networks has been introduced for identifying iRBD subjects using electroencephalography (EEG) data [9], achieving a 0.87 AUC. Machine learning (ML) models have been recently applied in many healthcare areas, including the neurological field, to support clinicians in the diagnosis of several neurological disorders. Indeed, ML models can capture complex, nonlinear relationships in medical data and learn the features in order to correctly identify different clinical phenotypes [10]. Several studies reported early Multiple Sclerosis (MS) diagnoses using ML algorithms [11]. Similarly, the excellent prediction performance of ML algorithms has inspired the development of novel models to identify the ischemic stroke [10]. Rechichi et al. [12] applied ML models to polysomnography features data in order to identify REM Sleep Without Atonia (RSWA), which is associated with RBD, obtaining an 87% accuracy. Another study [13] used Diffusion Tensor Imaging data to extract features and feed an SVM classifier in order to identify iRBD subjects with an 87.5% accuracy. The main limitation of these studies involves the complexity and cost of full EEG/EMG polysomnography and Magnetic Resonance Imaging (MRI) examinations, which make them unpractical for screening purposes.

To the best of our knowledge, there are no previous reports using an ML approach on HRV data to identify iRBD patients. Owing to this lack of investigation, here, we tested the accuracy of HRV features and ML models (Logistic Regression (LR), Extreme Gradient Boosting (XGBoost) and Random Forest (RF)) to correctly identify patients with iRBD.

## 2. Participants and Methods

### 2.1. Participants

Twenty patients with a clinical diagnosis of idiopathic RBD and twelve healthy, sex- and age-matched subjects (HC) were enrolled in this study (Table 1). All subjects underwent a complete neurological examination in order to exclude the presence of neurological disorders. All subjects underwent a video-polysomnography (PSG) to confirm or exclude the presence of clinical/subclinical RBD. In PSG recordings, a prominent muscle activity in REM sleep associated with abnormal behaviors was required to confirm the clinical or subclinical diagnosis of RBD [14] according to the International Classification of Sleep Disorders—Third Edition (ICSD-3). The current treatment with medications known to modify REM sleep architecture and muscle tone as serotonin reuptake inhibitors was considered as the exclusion criterion. Sleep stages were automatically scored on PSG recordings and manually checked by an expert scorer.

Controls were defined as having no history of neurological disorders and no clinical and polysomnographic confirmation of RBD. The presence of comorbidities known to affect the autonomic nervous system and interfere with autonomic evaluation was considered as the exclusion criterion for all participants.

All subjects gave written informed consent before participation. All the experimental procedures were conducted according to the policies and ethical principles of the Declaration of Helsinki. The study was approved by the Ethics Committee of the Calabria Region, “Sezione Area Centro” (no. 333, 22 October 2020).

### 2.2. Heart Rate Variability (HRV)

ECG traces were extracted from PSG recordings. Electrophysiological signals were sampled at 256 Hz. Filtered ECG signals were processed using the Hamilton Segmenter algorithm [15] for the identification of QRS complexes, and RR intervals were derived from R peaks sequences. RR intervals were then pre-processed for artifact removal and subdivided into 5 min windows, with a 50% overlap. Using sleep scoring from PSG, each window was tagged as “wake” (W) or “sleep” (S). The following HRV measures were extracted from each RR window [16]:

- NN/RR ratio: the fraction of total RR intervals that are classified as normal-to-normal (NN) intervals and included in the calculation of HRV statistics;
- AVNN: average of all NN intervals;
- SDNN: standard deviation of all NN intervals;
- rMSSD: square root of the mean of the squares of the differences between adjacent NN intervals;
- pNN50: percentage of differences between adjacent NN intervals that are greater than 50 ms;
- TOT_PWR: total spectral power of all NN intervals up to 0.04 Hz;
- VLF_PWR: total spectral power of all NN intervals between 0.003 and 0.04 Hz;
- LF_PWR: total spectral power of all NN intervals between 0.04 and 0.15 Hz;
- HF_PWR: total spectral power of all NN intervals between 0.15 and 0.4 Hz;
- LF/HF: ratio of low to high frequency power;
- Sample entropy of RR intervals;
- Largest Lyapunov exponent to quantify the amount of chaos in RR series;
- Hurst coefficient, as a measure of long-term memory in RR series;
- Alpha: scaling exponent from Detrended Fluctuation Analysis (DFA), for determining the statistical self-affinity of RR series;
- Triangular index: a geometric measure of HRV, defined as the integral of the density distribution (i.e., the number of all RR intervals) divided by the maximum of the density distribution;
- SD1: the standard deviation of the Poincaré plot perpendicular to the line-of-identity;
- SD2: the standard deviation of the Poincaré plot along the line-of-identity;
- SD2/SD1 ratio.

For each subject, the means of the above measures were computed for all S (sleep) windows and for the W (wake) windows from the last 15 min before falling asleep, when the subject is expected to be at rest. Moreover, for each measure, the sleep-to-wake ratio was evaluated. The means of the W and S segments and the ratios between the means of the S and W segments were tagged with “w”, “s” and “sw” suffixes, respectively. Therefore, a set of 54 features was derived for each subject.

In detail, for each subject, we calculated night-to-day ratios (cardiac autonomic indices) for both LF (cardiac sympathetic index) and HF (cardiac parasympathetic index) spectral components on a circadian cycle (24 h), as previously published [5]. According to our HRV protocol, all subjects stopped taking any anticholinergic, antidepressant, sympathomimetic or parasympathomimetic medications 72 h before testing and stopped taking levodopa 12 h before testing [5].

### 2.3. Machine Learning Approaches

A Receiver Operator characteristic (ROC) analysis was performed on single HRV features. Logistic Regression and ML models based on Random Forest (RF) [17] and eXtreme Gradient Boosting (XGBoost) [18] were trained on HRV data using Leave-One-Out Cross-Validation (LOO-CV) in order to evaluate classification performances in distinguishing iRBD from HC subjects. Feature importances were also evaluated, and, for each algorithm, several models were trained, grouping features according to their decreasing importance. For each model, training was performed using LOO-CV with the optimal number of features, ordered according to their decreasing importance, which produced the best classification accuracy. The hyperparameters of each ML model were tuned for optimal classification performances. Feature importances were evaluated as ROC AUC scores of single features, too. Such importances were used to choose the best discriminating feature and the best combination of features in order to train a logistic regression (LR) model, whose performances were assessed using LOO-CV.

ECG processing, R peaks extraction and preprocessing of RR intervals were performed in the Python programming language using SciPy, Nolds, hrv-analysis and BiospPy libraries. HRV features were calculated using the PhysioNet HRV Toolkit [19], a software tool written in the C programming language, after embedding it in Python code. ML analysis was performed using the *caret* package [20] in the R programming environment [21] (version 4.0.4, 2021, The R Foundation for Statistical Computing, Vienna, Austria).

## 3. Results

### 3.1. Features and HRV Analysis

There were no significant differences in data regarding gender and age between the two groups, as shown in Table 1. During 24 h registrations, we found a significant increase in autonomic indices in iRBD patients as compared to the controls (Table 1).

**Figure 1:**
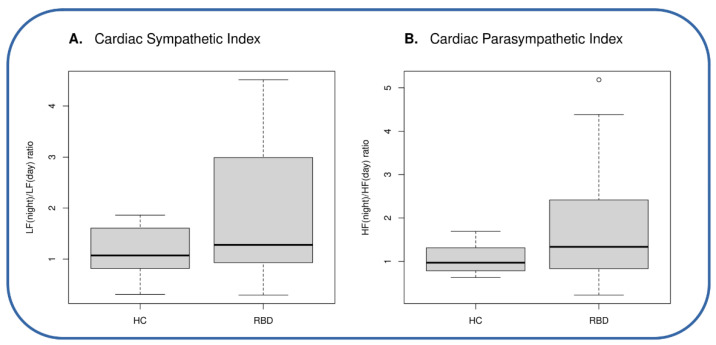
Box Plots of cardiac sympathetic (**A**) and parasympathetic (**B**) index in iRBD patients and controls. Each whisker extends to the most extreme data point (excluding outliers); the lower boundary of each box represents the first quartile, while the upper boundary identifies the third quartile; the bold horizontal line is the median value. Outliers are represented as small circles above or below whiskers. HC: Healthy Controls; iRBD: idiopathic REM Sleep Behavior Disorder; LF: power in the 0.04–0.15 Hz band; HF: power in the 0.15–0.4 Hz band.

In detail, at the cut-off levels of 2.06 and 1.38, which have been reported to accurately discriminate PD patients with and without RBD, both cardiac autonomic indices (cardiac sympathetic index and cardiac parasympathetic index, respectively) did not have a good classification performance, with the overlap of individual values between iRBD patients and controls (Figure 1A,B). Indeed, the accuracy of the autonomic indices was 0.63% for the sympathetic index and 0.69% for the parasympathetic index (Table 2)

### 3.2. Feature Importance and Feature Selection

**Figure 2:**
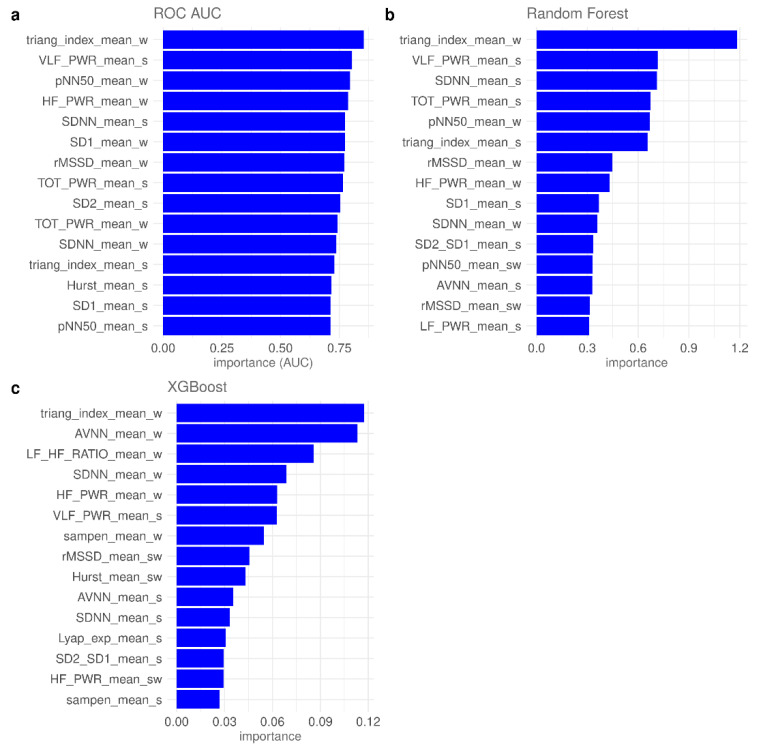
Feature importances evaluated from (**a**) ROC AUC and other ML models: (**b**) Random Forest and (**c**) XGBoost models.

Feature importances were evaluated using ROC AUC, Random Forest and XGBoost models, as shown in Figure 2a–c.

The Mean Triangular Index during wake (TIw) was identified as the best discriminating HRV feature using ROC AUC, and its classification performances were evaluated. The second best discriminating HRV variable was VLF power during sleep.

The five most important features evaluated by Random Forest in CARET were: wake triangular index, sleep VLF power, sleep SDNN, sleep total power and wake pNN50. XGBoost identified wake triangular index, wake AVNN, wake LF/HF ratio, wake SDNN and wake HF power as the five most important features using GLMnet. Though most important feature subsets evaluated by RF and XGBoost were different, both methods confirmed wake triangular index as the feature with the highest importance. TIw and sleep VLF power were identified as the two most important features by both ROC AUC and RF. Sex and age did not influence predictions, as they were ranked as the least important variables for discrimitating iRBD from HC subjects.

### 3.3. LOO-CV Results

**Figure 3:**
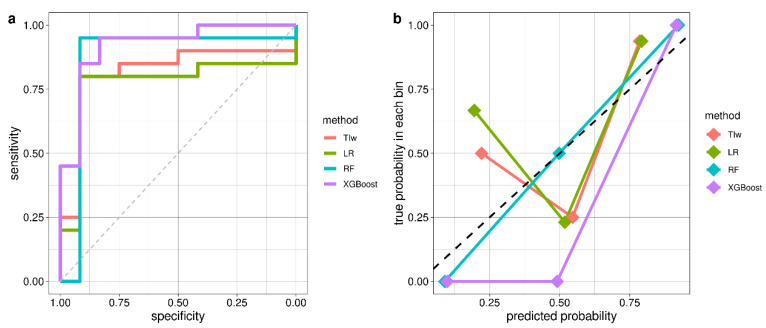
ROC curves (**a**) and calibration curves (**b**) from LOO-CV of TIw ROC (AUC = 0.82), Logistic Regression (AUC = 0.77) and optimal models with the best hyperparameters tuning: Random Forest (AUC = 0.87) and XGBoost (AUC = 0.92).

The ROC and calibration curves of each ML model trained using LOO-CV and the number of features, ordered according to their importance, which provided the best classification accuracy, are shown in Figure 3. The tuned hyperparameters of ML models are listed in Table S1 (Supplementary Materials).

Classification accuracies (with a 95% confidence interval), AUCs, sensitivities, specificities and the optimal number of variables for each ML model are reported in Table 3.

TIw has been identified as the feature that discriminated best between iRBD and HC, achieving a 0.81 accuracy and a sensitivity and specificity of 0.80 and 0.83, respectively. The LR model combining TIw and sleep VLF power slightly improved accuracy (0.84) by increasing specificity (0.92). The best classification accuracy (0.94) is achieved by the RF model trained using the five most important features (wake triangular index, sleep VLF power, sleep SDNN, sleep total power and wake pNN50; Figure 1A). The sensitivity and specificity were 0.95 and 0.92, respectively. The AUC corresponding to this optimal model, however, was lower than the classification accuracy. The best AUC (0.92) was reached by the XGBoost model trained on its 17 most important features (Figure 1B), with a 0.91 classification accuracy. With XGBoost, the specificity (0.83) was far lower than the sensitivity (0.95).

## 4. Discussion

To the best of our knowledge, this is the first study evaluating the potential of ML models for detecting patients with iRBD. Our study demonstrates that ML models applied on HRV features, accurately distinguished patients from controls. In particular, the RF algorithm showed the best classification performance, with an accuracy of 94%, followed by the XGBoost algorithm, with an accuracy 91%. By contrast, cardiac autonomic indices had lower classification performances in the differentiation between the two groups. It is also remarkable that both ML models independently identified iRBD with a high accuracy.

In our study, we built ML models on HRV parameters. HRV analysis has been widely used in movement disorders, and it represents one of the most promising quantitative markers of the cardiac autonomic balance. Indeed, HRV analysis has been demonstrated to be useful in the differentiation between ET patients and those with PD on an individual basis [22]. Changes in HRV parameters have also been observed during nocturnal sleep in patients with treated and untreated PD [23,24]. Interestingly, a previous study by our group has investigated the circadian autonomic change in HRV spectral components in PD patients associated with RBD compared to those with PD and without RBD during long-term conditions. We demonstrated that the night-to-day ratio of LF values (cardiac sympathetic index) accurately distinguished PD patients with RBD from those without RBD on an individual basis [5]. In the present study, we have also calculated the cardiac sympathetic and parasympathetic indices in patients and controls. However, we did not obtain the same classification performance, since the cardiac sympathetic index had an accuracy of 63% and the parasympathetic index had an accuracy of 69%. We believe that this discrepancy may be due to the fact that iRBD is really in the middle of a pathogenetic process in which many neuropathological alterations have already begun and others may occur. On this basis, the autonomic change in HRV parameters occurring in our iRBD patients could be less marked than those reported in a manifest phase of PD.

ML algorithms such as RF have been recently used to identify ischemic stroke by learning the features of the etiologies, and excellent classification performances were achieved [10]. Similarly, we used an RF model trained using the five most important features (wake triangular index, sleep VLF power, sleep SDNN, sleep total power and wake pNN50) to differentiate iRBD patients from controls. According to our results, the RF model showed a high accuracy (94%), while the AUC corresponding to this optimal model was lower than the classification accuracy, probably due to the small, imbalanced sample. We have also tested the accuracy of the Extreme Gradient Boosting (XGBoost) model trained on its 17 most important features. Indeed, XGBoost is an optimized combination of decision algorithms and linear regression analyses under a Gradient Boosting framework [11]. An early recognition model for MS based on XGBoost has recently been proposed [11]. According to these findings, in the training set, approximately 61%, 51% and 49% of the MS patients could be diagnosed with MS 1, 2 and 3 years earlier than their real diagnosis [11]. Of note, in our study, XGBoost showed a higher accuracy (91%), while the AUC was 92% and the classification accuracy was 91%. With XGBoost, the specificity (83%) was far lower than the sensitivity (95%), probably due to the imbalanced dataset. Finally, we have trained our ML models on HRV parameters obtained by electrocardiographic signals. However, we have previously demonstrated that there were small differences between the RR intervals evaluated with classic electrocardiography and pulse photoplethysmography [25]. In future investigations, we aim to also apply ML models on RR intervals obtained with pulse photoplethysmography.

There were some limitations to the study. First, the sample size of our cohort is small. Thus, a sample including more iRBD patients and controls is needed to validate the utility of ML models in clinical practice. Second, our ML model should be tested in other ethnic groups and regions, since it has been evaluated only in an Italian population. Finally, our target cohort included subjects suspected of having iRBD rather than the general population. Our study, however, has several strengths. First, our proposed ML models allowed for a correct identification of iRBD. Second, the artificial intelligence models have been trained on HRV features, simple and non-invasive measures that make this of particular practical value since it may be of valid help in the screening of patients suspected of having iRBD in large populations.

## 5. Conclusions

Our findings indicate that ML models applied on HRV features may help in distinguishing iRBD patients from controls. Our ML model could be easily implemented in a software application as a rapid screening tool, thus supporting neurologists in the early detection of this fascinating disorder.

## Figures and Tables

**Table 1:** Demographic and HRV data of enrolled subjects.

	HC	iRBD	*p* Value
N	12	20	--
Sex * (M/F)	7/5	14/6	0.70
Age ^#^ Sympathetic Index ^#^ Parasympathetic Index ^#^	54.2 ± 15.1 1.15 ± 0.52 1.05 ± 0.33	54.9 ± 9.1 1.86 ± 1.33 1.83 ± 1.42	0.89 0.04 0.03

* Fisher’s exact test. # Student’s *t*-test. Age, Sympathetic Index and Parasympathetic Index are expressed as the mean ± std.dev. HC: Healthy Controls; iRBD: idiopathic REM Sleep Behavior Disorder.

**Table 2:** Classification performances of cardiac autonomic indices.

Autonomic Indices	Accuracy (95% conf. int.)	AUC (95% conf. int.)	Sensitivity (95% conf. int.)	Specificity (95% conf. int.)
Sympathetic	0.63 (0.50–0.81)	0.62 (0.53–0.81)	0.45 (0.20–0.90)	1 (0.42–1)
Parasympathetic	0.69 (0.53–0.81)	0.65 (0.46–0.84)	0.55 (0.25–0.85)	1 (0.58–1)

AUC: Area Under the (ROC) Curve.

**Table 3:** Classification performances of ML models.

Model	Accuracy (95% conf. int.)	AUC	Sensitivity	Specificity	No. of Features
TIw	0.81 (0.64–0.93)	0.82	0.80	0.83	1
LR	0.84 (0.67–0.95)	0.77	0.80	0.92	2
RF	0.94 (0.79–0.99)	0.87	0.95	0.92	5
XGBoost	0.91 (0.75–0.98)	0.92	0.95	0.83	17

TIw: Mean Triangular Index (wake); LR: Logistic Regression; RF: Random Forest; XGBoost: eXtreme Gradient Boosting; AUC: Area Under the (ROC) Curve.

## Data Availability

The data presented in this study are available on request from the corresponding author. The data are not publicly available due to privacy restrictions.

## Supplementary Materials

The following supporting information can be downloaded at: https://www.mdpi.com/article/10.3390/diagnostics12112689/s1, Table S1: Best tuning parameters for ML models. R scripts and models, TRIPOD checklist.
