# Supplementary material for: A Machine Learning Approach for Detecting Idiopathic REM Sleep Behavior Disorder
Source: Diagnostics (Basel). 2022 Nov 4;12(11):2689. doi: 10.3390/diagnostics12112689 (PMC9689751; doi:10.3390/diagnostics12112689)
Supplement: Supplementary file 1 [file diagnostics-12-02689-s001.zip › informed_consent-module_HRV-RBD_study.pdf]

UMG dubium sapientiae initium UNIVERSITA' DEGLI STUDI "MAGNA GRÆCIA" CATANZARO

CENTRO DI RICERCHE NEUROSCIENZE

UNITA' DI RICERCA NEUROIMMAGINI CNR

RESPONSABILE: PROF. ALDO QUATTRONE

Consenso Informato

"Identificazione e validazione di nuovi biomarcatori, basati su tecniche di Intelligenza Artificiale applicate alla Variabilità della Frequenza Cardiaca, per la diagnosi precoce del Disturbo del comportamento in Sonno REM (RBD)"

(copia per il paziente)

Io sottoscritto/a (Nome e Cognome) ____

Dichiaro di aver ricevuto tutte le informazioni relative allo studio in oggetto, e di essere stato informato dal Prof.______________________________________ sugli scopi dello studio, sulle modalità attraverso le quali verrà effettuato e di aver avuto modo di chiedere ogni delucidazione.

Accetto volontariamente di partecipare allo studio sopraindicato.

Dichiaro altresì che mi sono state date tutte le informazioni necessarie per la comprensione del progetto a cui parteciperò.

Data _____________________ Firma______________________________________

- Dichiaro di aver ottenuto il consenso alla partecipazione allo studio del paziente/soggetto in ottemperanza alle disposizioni vigenti, alle norme di Buona Pratica Clinica ed ai principi della Dichiarazione di Helsinki e successive integrazioni e confermo di aver informato il soggetto secondo i contenuti prescritti dalle normative e secondo quanto stabilito nel protocollo sperimentale.

Nome e Cognome di chi ha informato la persona _________________________________

Data ________________ Firma di chi ha informato la persona_______________________

Consenso Informato

Studio

“Identificazione e validazione di nuovi biomarcatori, basati su tecniche di Intelligenza Artificiale applicate alla Variabilità della Frequenza Cardiaca, per la diagnosi precoce del Disturbo del comportamento in Sonno REM (RBD)”

(copia per il ricercatore)

Io sottoscritto/a (Nome e Cognome) ____

Dichiaro di aver ricevuto tutte le informazioni relative allo studio in oggetto, e di essere stato informato dal Prof.____ sugli scopi dello studio, sulle modalità attraverso le quali verrà effettuato e di aver avuto modo di chiedere ogni delucidazione.

Accetto volontariamente di partecipare allo studio sopraindicato.

Dichiaro altresì che mi sono state date tutte le informazioni necessarie per la comprensione del progetto a cui parteciperò.

Data ______________________ Firma_______________________________________

- Dichiaro di aver ottenuto il consenso alla partecipazione allo studio del paziente/soggetto in ottemperanza alle disposizioni vigenti, alle norme di Buona Pratica Clinica ed ai principi della Dichiarazione di Helsinki e successive integrazioni e confermo di aver informato il soggetto secondo i contenuti prescritti dalle normative e secondo quanto stabilito nel protocollo sperimentale.

Nome e Cognome di chi ha informato la persona _________________________________

Data ________________ Firma di chi ha informato la persona________________________
