# Supplementary material for: A Machine Learning Approach for Detecting Idiopathic REM Sleep Behavior Disorder
Source: Diagnostics (Basel). 2022 Nov 4;12(11):2689. doi: 10.3390/diagnostics12112689 (PMC9689751; doi:10.3390/diagnostics12112689)
Supplement: Supplementary file 1 [file diagnostics-12-02689-s001.zip › Supplementary Information.pdf]

Supplementary Material

Table S1. Hyperparameters tuning of Random Forest and eXtreme Gradient Boosting models

Model	Hyperparameters	definition
RF	Mtry = 2	Number of variables randomly sampled as candidates at each split
XGBoost	Nrounds = 3100	Maximum number of iterations/trees to grow
	Max.depth = 2	Depth of the tree
	Eta = 0.1	Learning rate
	Gamma = 0.05	Regularisation (preventing overfitting)
	Col.sample_by_tree=0.8	Controls the number of features supplied to a tree
	Child_weight = 1	Leaf threshold for stopping tree splitting
	Subsample = 0.5	Controls the number of samples supplied to a tree

RF: Random Forest; XGBoost: eXtreme Gradient Boosting.

Tuning of Hyperparameters for ML models is further described in the attached R code.
