# Supplementary material for: A Machine Learning Approach for Detecting Idiopathic REM Sleep Behavior Disorder
Source: Diagnostics (Basel). 2022 Nov 4;12(11):2689. doi: 10.3390/diagnostics12112689 (PMC9689751; doi:10.3390/diagnostics12112689)
Supplement: Supplementary file 1 [file diagnostics-12-02689-s001.zip › Tripod-Checlist-Prediction-Model-Development-HRV-ML-iRBD.pdf]

TRIPOD Checklist: Prediction Model Development

Section/Topic	Item	Checklist Item	Page
**Title and abstract**			
Title	1	A machine learning approach for detecting idiopathic REM Sleep Behaviour Disorder: a pilot study.	1
Abstract	2	Background and purpose: Growing evidence suggests that Machine Learning (ML) models can assist the diagnosis of neurological disorders. However, little is still known about the potential application of ML in diagnosing of idiopathic REM sleep behaviour disorder (iRBD), a parasomnia characterized by a high risk of phenoconversion to synucleinopathies. This study aimed to develop a model using ML algorithms to identify iRBD patients and test its accuracy. Methods: Data were acquired from 32 participants (20 iRBD patients and 12 controls). All subjects underwent a video-polysomnography. In all subjects, we measured the components of heart rate variability (HRV) during 24-hour recordings and calculated night-to-day ratios (cardiac autonomic indices). Discriminating performances of single HRV features were assessed. ML models based on Logistic Regression (LR), Random Forest (RF) and eXtreme Gradient Boosting (XGBoost) were trained on HRV-data. The utility of HRV features and ML models for detecting iRBD was evaluated by area under the ROC curve (AUC), sensitivity, specificity and accuracy corresponding to optimal models. Results: Cardiac autonomic indices had low performances (accuracy 63-69%) in distinguishing iRBD from control subjects. By contrast, RF model performed the best, with excellent accuracy (94%), sensitivity (95%) and specificity (92%), while XGBoost showed accuracy 91%, specificity 83% and sensitivity 95%. Mean triangular index during wake (TIw) was the best discriminating feature between iRBD and HC with 81% accuracy, reaching a 84% accuracy when combined with VLF power during sleep using a LR model. Conclusions: our findings demonstrated that ML algorithms can accurately identify iRBD patients. Our model could be used in clinical practice to facilitate the early detection of this form of RBD.	1
**Introduction**			
Background and objectives	3a	Idiopathic RBD (iRBD), and RBD in general, is currently diagnosed by means o polysomnography, which is expensive and may disturb the subject’s sleep. The RBD1Q questionnaire has been introduced as a screening tool, with high sensitivity (94%) and moderate specificity (87%). At present, no study has ever tried to identify iRBD using a Machine Learning (ML) approach on Heart Rate Variability (HRV) data.	2,3
	3b	This study aims at developing ML models for the identification of iRBD using HRV.	3
**Methods**			
Source of data	4a	Twenty patients with a clinical diagnosis of idiopathic RBD and twelve healthy, sex- and age-matched subjects (HC) were enrolled in this study	3
	4b	Start of enrollment: 01 Jan 2021<br>End: 31 Mar 2022	
Participants	5a	Secondary care unit, Neurosciences Research Centre, catanzaro, Italy.	3
	5b	iRBD: subjects reporting dream enactment (or reported by partners), with no other co-morbidities<br>Healthy Controls: no history of neurological disorders.	3
	5c	Medications modifying sleep architecture or autonomous nervous system activity were considered as exclusion criteria.	3
Outcome	6a	Presence/absence of iRBD.	4
	6b	NA. Outcome has to be compared to taget data.	4
Predictors	7a	HRV features, measured on adjacent 5-mins segments and averaged over sleep and wake periods	3-4
	7b	NA. Predictors have been chosen according to importance measures.	4
Sample size	8	Power analysis, considering a large effect (Cohen’s d=1.2). Initial calculations with n1=20 iRBD subjects suggested a number n2=8 HC subjects. Actual effect size of most important feature (TIw) was even larger (d=2.03).	
Missing data	9	No missing data.	
Statistical analysis methods	10a	AUCs and permutation importance of predictors were evaluated	4
	10b	Single feature (mean wake triangular index), Logistic Regression, Random Forest and extreme gradient boosting, using Leave-One-Out cross validation.	4
	10d	Accuracy, sensitivity, specificity, AUC	4
Risk groups	11	No risk groups.	
**Results**			
Participants	13a	Participants underwent a single polysomnographic examination with EEG, EMG, EOG and ECG recordings.	3
	13b	As described in the Methods section.	3
Model development	14a	20 subjects: outcome = 1<br>12 subjects: outcome = 0.	3
	14b	NA	

Model specification	15a	Models are included in the Supplementary Material.	
	15b	Prediction models are used as objects in R code, after evaluating new data/predictors from HRV data.	
Model performance	16	Mean wake Triangular Index: accuracy = 0.81 (0.64-0.93)<br>Logistic Regression: accuracy = 0.84 (0.67-0.95)<br>Random Forest: accuracy = 0.94 (0.79-0.99)<br>XGBoost: accuracy = 0.91 (0.75-0.98)	7
**Discussion**			
Limitations	18	The sample is small, though sufficient according to effect size calculations. For the validation of ML models a larger sample (from different ethnic populations) will be necessary, in order to introduce them into clinical practice.	8
Interpretation	19b	No other studies tried to identify iRBD using ML models on HRV features. Af HRV features can also be obtained far easily from photoplethysmografic (PPG) recordings, further investigation will be addressed to replicating these results on PPG HRV data.	8
Implications	20	Our proposed ML models allowed a correct identification of iRBD. Moreover, the artificial intelligence models have been trained on HRV features, simple and non-invasive measures that make this of particular practical value since it may be a valid help for the screening of patients suspected of having iRBD in large populations.	8-9
**Other information**			
Supplementary information	21	Trained ML models and R scripts are available in the Supplementary Material. Data are available upon reasonable request to the correspondig Author.	
Funding	22	No external funding has been received for this work.	

We recommend using the TRIPOD Checklist in conjunction with the TRIPOD Explanation and Elaboration document.
